# Cost-Effectiveness Analysis of Breast Cancer Control Interventions in Peru

**DOI:** 10.1371/journal.pone.0082575

**Published:** 2013-12-10

**Authors:** Sten G. Zelle, Tatiana Vidaurre, Julio E. Abugattas, Javier E. Manrique, Gustavo Sarria, José Jeronimo, Janice N. Seinfeld, Jeremy A. Lauer, Cecilia R. Sepulveda, Diego Venegas, Rob Baltussen

**Affiliations:** 1 Department of Primary and Community Care, Radboud University Nijmegen Medical Center, Nijmegen, the Netherlands; 2 Directorio Médico del INEN, Instituto Nacional de Enfermedades Neoplásicas (INEN), Lima, Peru; 3 Directorio Ejecutivo de Promoción de la Salud, Prevención y Control Nacional del Cáncer, Instituto Nacional de Enfermedades Neoplásicas (INEN), Lima, Peru; 4 Departamento de Radioterapia, Instituto Nacional de Enfermedades Neoplásicas (INEN), Lima, Peru; 5 Programa Comunitario de Salud Mamaria, PATH a catalyst for global health, Seattle, Washington, United States of America; 6 Centro de Investigación, Universidad Del Pacifico, Lima, Peru; 7 Costs, Effectiveness, Expenditure and Priority Setting, World Health Organization, Geneva, Switzerland; 8 Cancer Control, Management of Noncommunicable Diseases, World Health Organization, Geneva, Switzerland; 9 Dirección General de Salud de las Personas, Ministerio de Salud (MINSA), Lima, Peru; Boston Children's Hospital, United States of America

## Abstract

**Objectives:**

In Peru, a country with constrained health resources, breast cancer control is characterized by late stage treatment and poor survival. To support breast cancer control in Peru, this study aims to determine the cost-effectiveness of different breast cancer control interventions relevant for the Peruvian context.

**Methods:**

We performed a cost-effectiveness analysis (CEA) according to WHO-CHOICE guidelines, from a healthcare perspective. Different screening, early detection, palliative, and treatment interventions were evaluated using mathematical modeling. Effectiveness estimates were based on observational studies, modeling, and on information from Instituto Nacional de Enfermedades Neoplásicas (INEN). Resource utilizations and unit costs were based on estimates from INEN and observational studies. Cost-effectiveness estimates are in 2012 United States dollars (US$) per disability adjusted life year (DALY) averted.

**Results:**

The current breast cancer program in Peru ($8,426 per DALY averted) could be improved through implementing triennial or biennial screening strategies. These strategies seem the most cost-effective in Peru, particularly when mobile mammography is applied (from $4,125 per DALY averted), or when both CBE screening and mammography screening are combined (from $4,239 per DALY averted). Triennially, these interventions costs between $63 million and $72 million per year. Late stage treatment, trastuzumab therapy and annual screening strategies are the least cost-effective.

**Conclusions:**

Our analysis suggests that breast cancer control in Peru should be oriented towards early detection through combining fixed and mobile mammography screening (age 45-69) triennially. However, a phased introduction of triennial CBE screening (age 40-69) with upfront FNA in non-urban settings, and both CBE (age 40-49) and fixed mammography screening (age 50-69) in urban settings, seems a more feasible option and is also cost-effective. The implementation of this intervention is only meaningful if awareness raising, diagnostic, referral, treatment and basic palliative services are simultaneously improved, and if financial and organizational barriers to these services are reduced.

## Introduction

In Peru and other Latin-American countries, cancers have become a pressing health concern over the last decades. Cancer incidence and mortality rates have been rising and will probably continue to rise as a result of population growth, aging, urbanization and lifestyle changes [1-4].

In Peru, the highest cancer burden is currently represented by stomach, cervix, breast, prostate and lung cancer. Breast cancer is, together with cervical cancer, the leading cancer among females in terms of mortality and incidence [5,6]. Breast cancer has shown a persistent increase in incidence over the last decades, and many women present in advanced breast cancer stages. Efforts to control the disease in Peru are therefore essential (Table 1) [6,7].

**Table 1:** Age distribution of breast cancer incidence and mortality in Peru.

**Age groups**	Female population (2005)*	Incidence rate per 100.000)*	**Number of incident cases (%)**	Mortality rate (per 100.000)*	**Number of deaths (%)**	**Mortality / incidence ratio**	Current stage distribution (AJCC)**
**0-4**	1,382,448	0.0	0 (0%)	0.0	0 (0%)	n/a	**Stage I**
**5-14**	2,860,994	0.0	0 (0%)	0.0	0 (0%)	n/a	7.04%
**15-29**	3,801,363	1.28	49 (1.4%)	0.25	10 (0.5%)	0.20	**Stage II**
**30-44**	2,736,393	31.69	867 (24.2%)	9.66	264 (12.7%)	0.30	36.44%
**45-59**	1,654,473	85.79	1419 (39.6%)	46.22	765 (36.7%)	0.54	**Stage III**
**60-69**	630,326	85.17	536 (15.0%)	64.45	406 (19.5%)	0.76	43.48%
**70-79**	400,815	121.59	487 (13.6%)	104.57	419 (20.1%)	0.86	**Stage IV**
**80+**	142,471	158.61	226 (6.3%)	153.32	218 (10.5%)	0.98	13.04%

* WHO Global Burden of Disease, 2004 update [7].

** INEN 2007-2011[12].

As a public response, the Peruvian Ministry of Health (MINSA) and allied institutions developed multi-sectoral cancer control strategies in 2006, focusing on prevention, education, early detection and expanding services for multiple cancers [8]. The strategic program, explicitly for breast cancer, consists of group and individual counseling in breast cancer prevention (women aged 18 to 64 years) as well as the promotion of annual mammography screening (age group 40-65). Furthermore, with the goal of reaching universal coverage, the Peruvian government introduced a universal health insurance law in 2009 and has also gradually been devoting more financial resources to cancer control [9-11].

Despite these developments, the implementing institutions face significant problems with the roll out of cancer control strategies. The coverage of breast cancer services is only partial and unequal, partly due to a fragmented health system, decentralization, and the unguaranteed financial resources [12]. In addition, only 51.8% of the population was insured (INEI 2008) and breast cancer treatment and rehabilitation, only partially covered by insurance, may require substantial out of pocket payments [13]. This may lead to important financial, cultural and geographical disparities in access to breast cancer care for many Peruvian women [14].

With this background, and with the rising cost of cancer control to the Peruvian healthcare system, MINSA is facing difficult choices on which breast effective cancer interventions to provide and at which cost they can be sustained for the long term. Also, given its limited health-care resources, Peru needs to spend money wisely and fund interventions that provide best value for money. Cost-effectiveness analysis (CEA) is a tool that systematically compares costs and effects of health interventions and that can guide policy makers in these decisions. Results from CEAs can for example be used to improve the general planning of strategies, or to strengthen certain breast cancer strategies by demonstrating their value for money. So far, CEAs on breast cancer interventions have not been conducted in Peru [15], hence, important information on efficient breast cancer control strategies is currently lacking.

To support breast cancer control in Peru, this study aims to explore and report the cost-effectiveness of different breast cancer control interventions relevant for the Peruvian context. In this paper, we provide an outline of the most efficient and feasible interventions for breast cancer control in Peru from the healthcare perspective.

## Methods

### General approach

Our standardized CEA methods, derived from WHO-CHOICE, are described in detail elsewhere and build on previous regional and country specific analyses of interventions to control breast cancer [16-20]. An important feature of this methodology is that all possible interventions are compared to a situation where no interventions are implemented. This counterfactual acts as a reference to compare the cost and effects of all possible interventions, and enables us to make comparisons across a wide range of competing interventions (e.g. tuberculosis, mental disorders, non-communicable diseases) [16,21-24].

### Breast cancer data and interventions

To select a set of breast cancer interventions relevant to Peru and to identify sources for cost and effectiveness data, a local study team was established during a stakeholder meeting in 2011. The team, consisting of representatives from the Ministry of Health (MoH), the national cancer institute (INEN), the social security network (EsSalud), PATH, and the World Health Organization (WHO/PAHO), could propose any type of breast cancer intervention.

An assessment tool, to collect information on breast cancer programs and their coverage, finance, and epidemiology, was developed by the WHO and sent to the study team leader (INEN). Its results provided key data for our analyses and an overview of the current breast cancer activities in Peru [12].

From a standard set of breast cancer control interventions [17,18], the study team identified a set of 15 interventions relevant to in the Peruvian context, all related to breast cancer treatment, early diagnosis, screening or palliative care (Table 2). The study team introduced a particular intervention of interest, relating to the diagnostic procedure of women with palpable masses detected through clinical breast examination (CBE) screening [25,26]. This intervention aims at improving the capacity of early breast cancer diagnosis (i.e. confirmation by triple test) during CBE screening, by using upfront fine needle aspiration (FNA) first - instead of mammography first - at the primary healthcare level (Figure S1). In this way, the number of (technically more demanding) mammograms and core biopsies at the primary healthcare level could be reduced. The study team also adjusted standard treatment regimes of previous WHO-CHOICE analyses, and added therapies for HER2neu positive women (Trastuzumab). Furthermore, various combinations of screening age groups (40-69/40-64/45-69/45-64/50-69/50-64 years) and screening frequencies (annually/biennially/triennially) were introduced for the screening interventions. Additionally, the team defined different screening interventions specifically for rural areas (CBE screening vs. mobile mammography units in 40% of the total population) and urban areas (e.g. only fixed mammography units in 60% of the total population) according to the Peruvian urbanization rate (60%).

**Table 2:** Definition and classification of selected interventions for breast cancer control in Peru.

**Treatment of individual stages**	**#**	**Awareness raising** †	**#**	**Screening** †	**#**	**Palliative Care‡**	**#**
**Stage I treatment**: lumpectomy with axillary dissection and radiotherapy (33 fractions).* Eligible patients receive tamoxifen** [18,60]	**#4**	**Basic Awareness Raising (BAR**): community nurses training program + opportunistic outreach activities by community nurses to raise breast cancer awareness and educate on breast self examination techniques (BSE) + enhanced media activities [37].	**#13**	**CBE screening: Clinical breast examination (CBE**)** screening (95% coverage**) in asymptomatically women: community nurses training program + active outreach screening by community nurses + limited media and awareness raising activities [53]. ages 40-69/40-64/45-64/45-69/50-69/50-64 annual/biennial/triennial	**#15-32**	**Standard Palliative Care (SPC**): pain treatment through pain medication and anti-emetics, palliative radiotherapy (8 Gy in 1 boost) for eligible patients. Includes end of life hospitalization. No home based visits[60,61].	**All except #11-12, 91-94**
**Stage II treatment**: lumpectomy with axillary dissection (70%), or modified radical mastectomy (30%) followed by adjuvant chemotherapy*** and radiotherapy (33 or 25 fractions)* Eligible patients receive tamoxifen** or chemotherapy. ‡ [18,60]	**#5**	**Mass-media awareness raising (MAR**): BAR + mass media campaign (weekly emissions) [37]	**#14**	**Mammography fixed screening urban only : Mammography screening urban (57% coverage by fixed mammography units**) in asymptomatic women + limited media and awareness raising activities [17]. ages 40-69/40-64/45-64/45-69/50-69/50-64 annual/biennial/triennial	**#33-52**	**Basic Palliative Care (BPC**): SPC + palliative care-volunteers training program + home based visits by volunteers every fortnight. Includes end of life hospitalization [60-62].	**#11, 91**
**Stage III treatment**: modified radical mastectomy followed by adjuvant chemotherapy*** and radiotherapy (25 fractions).* Eligible patients receive tamoxifen.** [18,60]	**#6**			**Mammography screening fixed (urban**)** and mobile (rural**): Mammography screening fixed (57% coverage by fixed mammography units + 38% coverage by mobile units) in asymptomatic women + limited media and awareness raising activities [17]. ages 40-69/40-64/45-64/45-69/50-69/50-64 annual/biennial/ triennialages 40	**#53-70**	**Extended Palliative Care (EPC**): SPC+ BPC apart from community nurses instead of palliative care-volunteers, medication strengthened with anti-depressants, and bisphosphonates. Includes end of life hospitalization [60-63].	**#12, 92-94**
**Stage IV treatment**: adjuvant chemotherapy*** and radiotherapy (10 whole +3 boost fractions) + Standard Palliative Care. Eligible patients receive tamoxifen **[18,60]	**#7**			**Mixed Screening:** **Urban: Mammography screening** urban (57% coverage by fixed mammography units) only **in ages >50** / combined with **CBE screening in ages <50** urban (57% coverage) in asymptomatic women + limited media and awareness raising activities[17]. **Rural: CBE screening all ages** in non-urban areas (38% coverage) in asymptomatically women: community nurses training program + active outreach screening by community nurses + limited media and awareness raising activities[53]. ages 40-69/40-64/45-64/45-69/50-69/50-64 annual/biennial/ triennial	**#71-88**		
**Stage I to IV combined** without trastuzumab with trastuzumab in all HER2 positives (stage I to IV).with trastuzumab in early stage HER2 positives (stage I and II only)	**#8-10**			**Upfront FNA (fine needle aspiration**)** after a positive CBE screen, only in combination with CBE screening**: FNA training program for GP/medical officer at district hospitals + training of cytologists (2 per province/year). FNA samples are evaluated at district level, and eligible patients referred to provincial or national hospitals[38]. Combined only with the most cost effective biennial and triennial CBE screening intervention	**#33-34, 89-93**		

* Radiotherapy generally includes a dose of 50 Gy given in 10-33 fractions or boosts on an outpatient basis.

** Endocrine therapy consists of 20 mg. tamoxifen per day for 5 years.

*** The (neo)adjuvant chemotherapy combination regimen consists of AC-Taxol: AC given 3-weekly for 4 cycles followed by paclitaxel given weekly for 12 weeks.

^†^ Down-staging interventions cause a shift in stage distribution and are only modeled in combination with treatment of all stages (I to IV).

^‡^ Palliative care interventions BPC and EPC are only applied to stage IV patients, and substitutes Standard Palliative Care.

^#^ Scenario number in supplement (Table S2) and Figure 2.

We combined the 15 interventions to construct a total of 94 intervention scenarios. This includes the current Peruvian situation in which patients of stages I to IV are treated at a 50% coverage level, along with preventive counseling (30% coverage) and opportunistic screening (15% coverage)[12]. Other interventions are evaluated at a geographic coverage level of 60%, 80% or 95% (i.e. reaching 95% of those who need services) according to standard CHOICE methodology.

### Mathematical model

**Figure 1:**
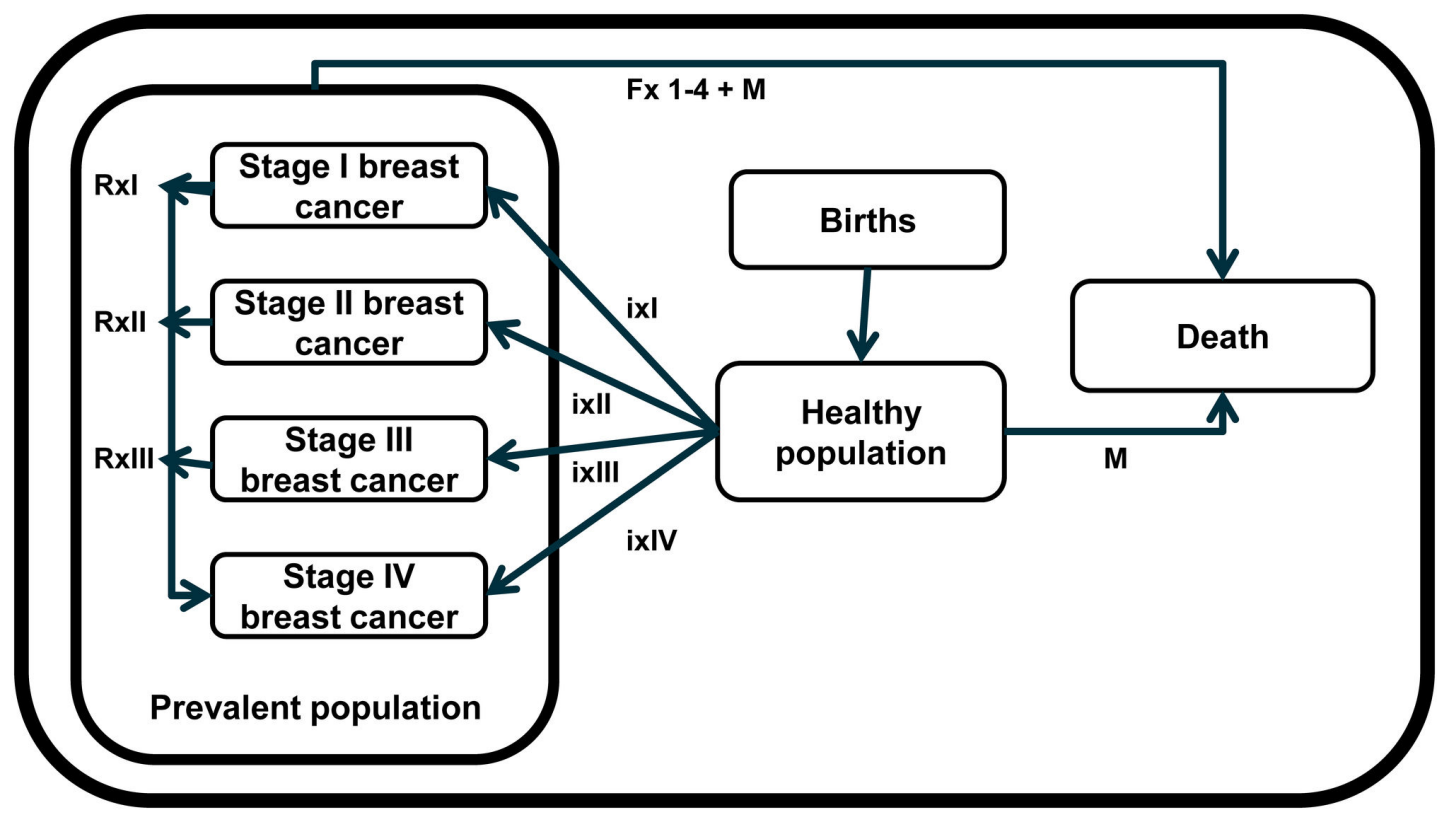
Graphical representation of the model. Graphical representation of the model showing the relationships between the different health states through the incidence rates of breast cancer (Ix1–Ix4), the different stage specific case fatality rates (Fx1–4), and the background mortality (M). Stage specific relapse rates to stage IV were used to correct the disability weights (Rx1–Rx3).

The model structure is presented in Figure 1 and includes a healthy state, a deceased state, and stage I to IV breast cancer states following the American Joint Committee on Cancer (AJCC) [18,27]. To assess health outcomes, WHO-CHOICE employs an epidemiological, population based approach. The national breast cancer epidemiology is entered into a state transition model, along with background birth, population, and mortality rates, to estimate the total number of disability adjusted life years (DALYs) experienced over the lifetime (100 years) of the Peruvian population [28]. The effectiveness of interventions is expressed in changes in case fatality (treatment interventions), health state valuations (HSVs), or stage distribution (awareness raising and screening interventions). Interventions are taken to be implemented for a period of 10 years, after which epidemiological rates go back to their counterfactual level of no intervention. The difference in the total number DALYs lived by the population between each scenario and the null-scenario gives the population health gains in DALYs averted. Consistent with the WHO Global Burden of Disease study, DALYs are discounted (at 3% per year) and age weighted.

### Effectiveness data

We based epidemiological data on the WHO Global Burden of Disease (GBD) study, applied to the population of Peru of 2011 [5,7,29]. The impact of treatment in Peru was estimated on the basis of stage specific survival rates (case-fatality) from INEN (2000-2010) and previous WHO-CHOICE analyses [18,30], whereas the impact of trastuzumab on case fatality was based on the literature (Table 3) [31]. 

**Table 3:** Case fatality rates, disability weights and stage distribution used for intervention combinations in Peru.

**Intervention**	Case fatality rates*				Disability weights**				Stage distribution***			
	***Stage I***	***Stage II***	***Stage III***	***Stage IV***	***Stage I***	***Stage II***	***Stage III***	***Stage IV***	***% in stage I***	***% in stage II***	***% in stage III***	***% in stage IV***
Untreated	0.021	0.065	0.156	0.311	0.086	0.097	0.104	0.375	7.0%	36.4%	43.5%	13.0%
Treatment only	0.006	0.040	0.093	0.275	0.086	0.097	0.104	0.154	7.0%	36.4%	43.5%	13.0%
Treatment only + Trastuzumab in all HER2 positives	0.006	0.038	0.086	0.247	0.086	0.097	0.104	0.154	7.0%	36.4%	43.5%	13.0%
Current country specific situation (50% coverage), annual opportunistic screening (15%) and free consultation (30%)	0.006	0.040	0.093	0.275	0.086	0.097	0.104	0.153	7.0%	36.4%	43.5%	13.0%
Basic Palliative Care (BPC)	0.006	0.040	0.093	0.275				0.0153				13.0%
Extended Palliative Care (EPC)	0.006	0.040	0.093	0.275				0.0152				13.0%
Basic Awareness Raising (BAR)	0.006	0.040	0.093	0.275	0.086	0.097	0.104	0.154	10.2%	20.1%	44.8%	24.8%
Mass media Awareness Raising (MAR)	0.006	0.040	0.093	0.275	0.086	0.097	0.104	0.154	21.1%	41.5%	24.1%	13.3%
Annual CBE screening (age 40-69/40-64/45-64/45-69/50-69/50-64)	0.006	0.040	0.093	0.275	0.086	0.097	0.104	0.154	29.2%-15.8%	31.2%-16.9%	30.4%-51.5%	9.3%-15.8%
Biennial CBE screening (age 40-69/40-64/45-64/45-69/50-69/50-64)	0.006	0.040	0.093	0.275	0.086	0.097	0.104	0.154	26.9%-14.0%	28.8%-14.9%	33.9%-54.4%	10.4%-16.7%
Triennial CBE screening (age 40-69/40-64/45-64/45-69/50-69/50-64)	0.006	0.040	0.093	0.275	0.086	0.097	0.104	0.154	25.4%-12.8%	27.2%-13.7%	36.3%-56.2%	11.1%-17.2%
Annual mammography screening FIXED 60% (age 40-69/40-64/45-64/45-69/50-69/50-64)	0.006	0.040	0.093	0.275	0.086	0.097	0.104	0.154	26.2%-19.7%	29.7%-22.7%	33.6%-43.9%	10.5%-13.7%
Biennial mammography screening FIXED 60% (age 40-69/40-64/45-64/45-69/50-69/50-64)	0.006	0.040	0.093	0.275	0.086	0.097	0.104	0.154	25.7%-19.0%	29.1%-22.0%	34.4%-44.9%	10.8%-14.0%
Triennial mammography screening FIXED 60% (age 40-69/40-64/45-64/45-69/50-69/50-64)	0.006	0.040	0.093	0.275	0.086	0.097	0.104	0.154	25.2%-18.6%	28.6%-21.5%	35.1%-45.7%	11.0%-14.3%
Annual mammography screening FIXED/MOBILE (age 40-69/40-64/45-64/45-69/50-69/50-64)	0.006	0.040	0.093	0.275	0.086	0.097	0.104	0.154	37.4%-26.5%	40.0%-28.4%	17.3%-34.5%	5.3%-10.6%
Biennial mammography screening FIXED/MOBILE (age 40-69/40-64/45-64/45-69/50-69/50-64)	0.006	0.040	0.093	0.275	0.086	0.097	0.104	0.154	36.5%-25.4%	39.0%-27.2%	18.8%-36.2%	5.7%-11.1%
Triennial mammography screening FIXED/MOBILE (age 40-69/40-64/45-64/45-69/50-69/50-64)	0.006	0.040	0.093	0.275	0.086	0.097	0.104	0.154	35.8%-24.6%	38.3%-26.4%	19.9%-37.5%	6.1%-11.5%
Annual CBE/mammography screening MIXED (age 40-69/40-64/45-64/45-69/50-69/50-64)	0.006	0.040	0.093	0.275	0.086	0.097	0.104	0.154	33.6%-22.3%	36.0%-23.8%	23.3%-41.3%	7.1%-12.6%
Biennial CBE/mammography screening MIXED (age 40-69/40-64/45-64/45-69/50-69/50-64)	0.006	0.040	0.093	0.275					32.1%-20.9%	34.3%-22.3%	25.7%-43.5%	7.9%-13.3%
Triennial CBE/mammography screening MIXED (age 40-69/40-64/45-64/45-69/50-69/50-64)	0.006	0.040	0.093	0.275	0.086	0.097	0.104	0.154	31.0%-19.9%	33.2%-21.3%	27.4%-45.0%	8.4%-13.8%

Current country specific situation: Current situation in Peru with treatment coverage of 50%, annual opportunistic screening (15%) and free preventive consultations (30%)[12].

* Derived from Bland et al. and stage I and II corrected for the addition of chemotherapy [30]. For trastuzumab CFs were multiplied with 0.66 [31]for eligible patients (eligibility = 12.7% stage I, 12.07%, stage II, 22.0%, stage III, 30.4% stage IV) [64].

** The DW for stage I is equal to the GDB estimate, while for other stages the GBD long term sequel (0,09) was adjusted according to utilities from the literature [7,32,33] and corrected for relapse to stage IV. Relapse rates were derived from Adjuvant Online [65].

*** Present stage distribution is based on INEN public sector [12]. Effects of MAR derived from Devi et al.[37] Effects of screening interventions were based on stage shifts from baseline [17] to the stage distribution in The Netherlands[35]. Stage shifts were adapted by calculating relative differences in detection rates between The Netherlands and Peru[34]. Calculations included age specific incidence, prevalence [7], sojourn time[34], sensitivity [36] and attendance rates (72% in Peru).

Health state valuations originate from disability weights (DWs) of the GBD study, and we assumed that interventions affect DWs in stage IV only. The DW for stage I is equal to the GDB estimate, while for other stages the GBD long term sequel (0,09) was adjusted according to quality of life estimates from the literature [32,33]. The current stage distribution of women presenting with breast cancer in Peru was derived from INEN (2007-2011)[12], and the impact of the various screening interventions on this stage distribution was estimated on the basis of a model following Duffy et al. by using proportional detection rates [34]. We applied a stage shift from developing countries [17] to the Dutch screening program [35], and corrected this shift for locally relevant attendance rates (72%) and the Peruvian epidemiology and demography. The age specific sensitivity of tests and sojourn times (CBE sojourn times are two-third that of mammography) were based on the literature [34-36]. The effectiveness of the awareness raising interventions are based on a study from Malaysia [37] while we assumed a twofold effect on stage distribution when applying a mass media campaign.

### Cost data

Following standardized WHO-CHOICE methodology on CEA, we used an ingredients approach for our costing analysis, in which prices and quantities are separately reported. We distinguished patient-level and program-level costs, and to estimate the total patient costs of interventions we multiplied the unit costs of patient services with the number of patients requiring these services.

Unit costs of patient services were based on the principles of micro-costing, including detailed resource utilization patterns and prices for each procedure (Table S1, Table 4). INEN provided these unit cost to a great level of detail, except for the cost of facilities (buildings, rooms) and the cost for the transportation of drugs and supplies. We derived the transportation multipliers, the size, price and annualization factors for facilities, from a standard WHO-CHOICE database and applied them to each eligible item [20]. 

**Table 4:** Average utilization of main diagnostic and treatment services and unit costs per patient.

**Procedure**	**Ingredients**	**Stage I**	**Stage II**	**Stage III**	**Stage IV**	**Palliative** (**SPC**)†	**Unit cost per patient (US$)**
**Initial diagnosis and evaluation during treatment**	Medical consultation	2	2	2	2		6.22
	Core biopsy procedure	1	1	1	1		45.02
	Specimen examination	1	1	1	1		9.76
	Bilateral Mammography	1	1	1	1		14.24
	Echo of breast	1	1	1	1		6.20
	Echo of abdominal/pelvic area	1	1	1	1		10.49
	Liver function tests	1	1	1	1		2.07
	Chest X-ray	1	1	1	1		6.79
	Bone scan	1	1	1	1		46.01
	CT of chest	1	1	1	1		96.37
	CT of abdominal/pelvic area	1	1	1	1		115.50
	Multidisciplinary consult	1	1	1	1		100.90
**Treatment**	Pre-operative tests	1	1	1	-		86.57
	Surgical risk analysis	1	1	1	-		20.18
	Surgery	1 (lumpectomy)	1 (lumpectomy/modified radical mastectomy)	1 (modified radical mastectomy)	-		835.88 / 951.77
	Radiotherapy consult	1	1	1	1		7.64
	Radiotherapy planning & first administration*	1	1	1	1		224.20
	Radiotherapy session administration*	32	29.6	24	12		23.36
	AC regimen**	-	4	4	4		104.00
	Taxol regimen**	-	12	4	4		134.47
	Hepatic tests	-	12	12	12		22.14
	Renal tests	-	12	12	12		39.38
	Coagulation tests	-	12	12	12		115.40
	CT	-	2	4	4		115.50
	Bone scan	-	2	2	2		46.01
	% receiving endocrine treatment***	1680	1680	336	336		0.18
	% receiving pain medication					1	9136.87
	% receiving emetics					1	1903.52

* Radiotherapy generally includes a dose of 50 Gy given in 10-35 fractions or boosts on an outpatient basis.

** The (neo) adjuvant chemotherapy combination regimen consists of AC-Taxol: AC given 3-weekly for 4 cycles followed by paclitaxel given weekly for 12 weeks or 4 weeks.

*** Endocrine therapy consists of 20 mg. tamoxifen per day for 5 years.

^†^ Palliative care is only applied to stage IV patients. Standard Palliative Care (SPC) does not include home based visits. Medication includes Tramadol 50 ml, Morphine 0.02 mg, Fentanyl 50 mg, Parecoxib 40 mg, Triamcinolone 50 mg, Diazepam, Lidocaine, epidural injections, Omeprazol 40 mg, Haloperidol 5mg, Levosulpiride 25mg.

We estimated the costs for the FNA intervention through a patient management scheme from the international literature, as this data was not yet available in Peru [38]. We then used average weighted resource patterns for FNA, based on observational studies from different countries [39-44], and assumed similar final outcomes for both CBE screening with upfront FNA and usual CBE screening (Figure S1). 

Program-level costs capture management, administrative, media and law-enforcement costs, and costs for training of healthcare personnel. These costs were based on estimates from WHO-CHOICE and from Peruvian program managers (INEN). Media and operating costs (i.e. prices for broadcasting, flyers, and posters) were based on local inventories of prices, also provided by INEN. 

For all interventions, we also included costs of diagnostic tests for women presenting with initial symptoms without breast cancer (true-negatives), and assumed the ratio of tested negatives *vs.* tested positives to be 16.4:1 in non-screened populations and 21.5:1 in screened populations [45,46]. Single treatment scenarios also include the costs of diagnosing all other stages, and regarding screening interventions, we included costs for evaluating false positives [47].

All costs were estimated in 2012 Peruvian Soles and converted to U.S. dollars (US$) using the 2012 exchange rate (1US$ = 2,603SOL). Both health effects (DALYs) and costs (US$) were discounted at an annual rate of 3%. 

### Cost-effectiveness analysis

The average cost effectiveness ratio (ACER) for each intervention is calculated by dividing the total costs of an intervention by its corresponding effects, relative to the comparator situation of no intervention. 

In addition to these ACERs, incremental cost effectiveness ratios (ICERs) are reported for the successive set of interventions that can be purchased at expanding levels of resource availability, starting with the intervention with the lowest cost per DALY averted, then moving to the next most cost-effective intervention. The order in which interventions can be selected according to their ICER is called an expansion path, and only interventions that are both more effective and less costly than other (combinations of) interventions are considered on this expansion path. The incremental cost-effectiveness ratios (ICERs) for those interventions are calculated by dividing the incremental costs by the incremental health effects.

CEA results should be furthermore interpreted according to a defined set of cost-effectiveness thresholds. WHO-CHOICE denotes an intervention as “cost-effective” if it produces a healthy year of life for less than three times the gross domestic product (GDP) per capita, and as “very cost-effective” if it produces a healthy year of life for less than the GDP per capita (human capital approach) [48]. In Peru, this means that interventions that cost less than $4,608 per DALY averted can be considered very cost-effective, and interventions that cost between $4,608 and $12,204 can be considered as cost-effective.

### Sensitivity Analysis

We performed a deterministic sensitivity analysis to assess the robustness of the results to potential changes in key assumptions regarding the model parameters. Based also on the results of previous sensitivity analyses [16-18], the baseline case fatality rates and DWs were varied +/- 25% and we used other sources for Peru’s current case fatality rates [12] and current stage distribution [49]. The effect of awareness raising interventions was reduced by 25%, and we lowered attendance rates of screening interventions and the sensitivity of CBE and mammography tests (-25%). Regarding costs, we varied the transportation multipliers (+/- 25%), and varied the unit costs of CBE and mammography (+/- 25%) as well as the costs for FNA (+/- 25%).

## Results

**Figure 2:**
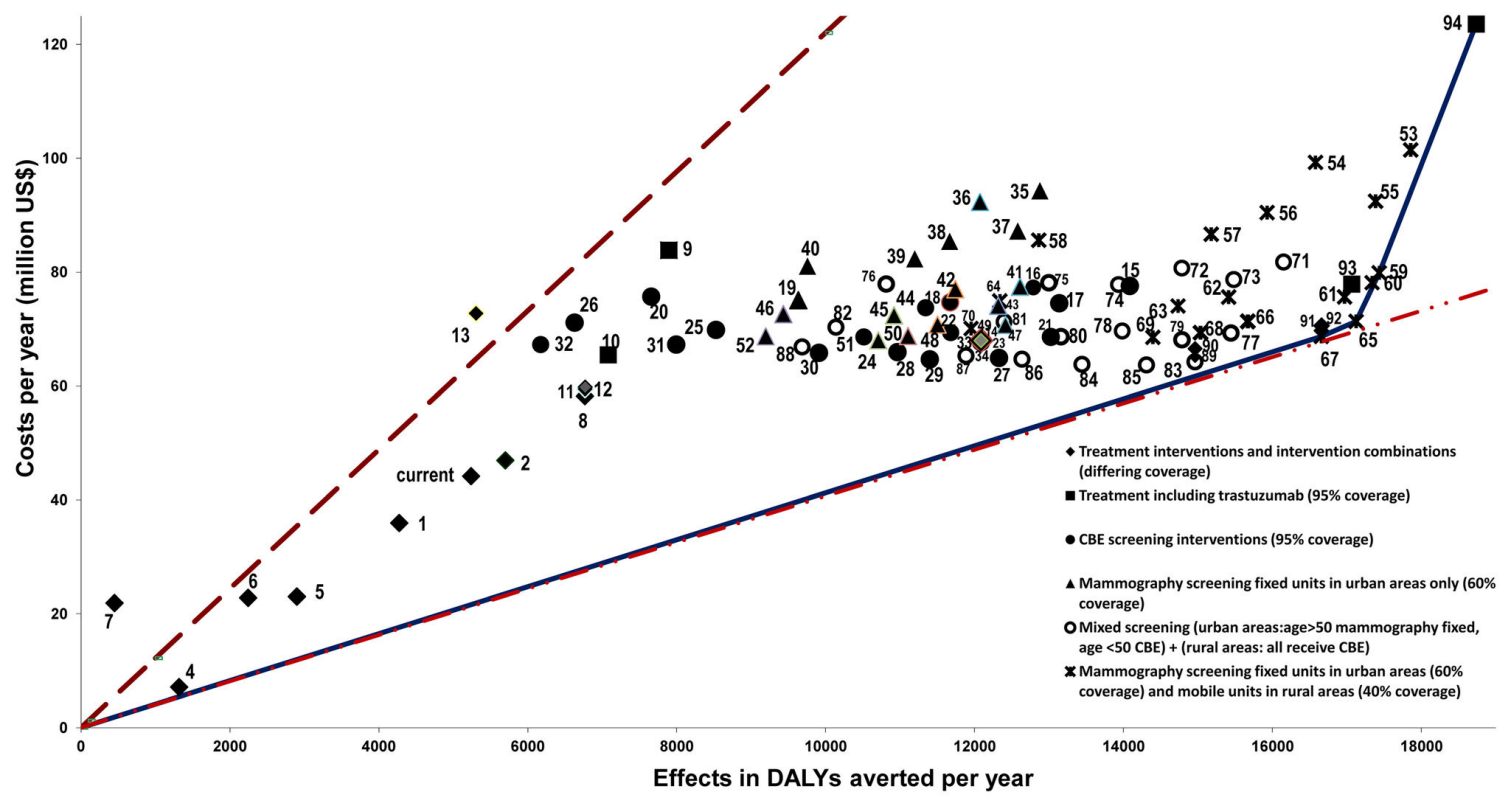
Cost-effectiveness frontier.

A total of 94 single and combined intervention strategies were assessed and their annual cost, effects, and cost-effectiveness are provided in Table S2 and shown graphically in Figure 2.

Cost-effectiveness of breast cancer interventions and expansion path according to ICER (Incremental cost-effectiveness ratio). Dotted lines represent the cost-effectiveness threshold of 3*GDP/capita/DALY averted (12.204 US$/DALY) and 1* GDP/capita/DALY (4.068 US$/DALY).The annual treatment costs for breast cancer stages I to IV, vary between 7.1 million (treatment of stage I) and 23.0 million US$ (treatment of stage II). Treatment of all stages costs more than $58 million (95% coverage) and expanding this by providing trastuzumab in all stages costs an extra $25 million ($83.8 million in total), while the additional cost for providing trastuzumab only in stage I&II is about $7 million ($65.4 million in total). The additional costs for providing basic or extensive palliative are $1.2 million and $1.6 million ($44.4 and $59.5 million in total) respectively, whereas awareness raising interventions cost $59.8 million (BAR) and $56.0 million (MAR). 

The costs of screening interventions generally increase with the screening frequency, e.g. in age groups 40 to 69, the costs are $74.3 to $101.5 million for annual screening strategies and $63.4 to $71.4 for triennial screening strategies. Furthermore, when age group 40 to 45 is included in the screening strategy or annual screening frequencies are applied, costs increase relatively more as compared to age group 65 to 69 and triennial screening frequencies. Screening costs also increase when a mobile screening component is applied, i.e. the costs per mammogram or CBE with a mobile unit are about 20% higher.

The upfront FNA component reduces the costs of CBE screening (as compared to the usual CBE screening strategy) by an estimated $3.48 per women diagnosed (Figure S1). As a result, CBE screening interventions combined with upfront FNA have slightly lower patient costs but higher program and training costs.

In the individual stage I to IV treatment interventions the annual number of DALYs averted vary between 451 (stage IV) and 2,900 (stage II). Jointly these interventions can avert 6,757 DALYs. The addition of trastuzumab to eligible women in stage I&II only averts 313 extra DALYs (a total of 7,080 DALYs), whereas providing trastuzumab to eligible women in all stages can avert 1128 extra DALYs (a total of 7,895 DALYs). The addition of palliative care, both basic (BPC) and extended (EPC), only adds very few DALYs. Awareness raising interventions, combined with treatment of all stages and standard palliative care, avert between 5,306 (BAR) and 12,115 DALYs (MAR). 

The various screening intervention combinations avert between 6,000 and 18,000 DALYs, generally much more than treatment interventions only. By increasing screening frequencies and by widening age groups, screening interventions can avert more DALYs. With regards to the age group of screening, including the oldest age group (65-69) seems to avert relatively more DALYs as compared to including the youngest age group (40-45).

Annual screening through fixed (urban) and mobile (non-urban) mammography units has the most health impact, and can avert more than 18,000 DALYs when applied in age group 40 to 69 and combined with EPC.

Average cost-effectiveness ratios (ACERs) of the individual treatment interventions range between $5,406 (stage I treatment) and $48,5676 (stage IV treatment) per DALY averted. Treatment of all stages costs $8,605 per DALY averted costs around $10,000 per DALY averted when trastuzumab is added. Palliative care interventions costs $8,782 (BPC) and $8,832 per DALY averted (EPC).

With regards to current breast cancer program in Peru, the ACER of this scenario (scenario 3) is $8,426 per DALY averted. The ACER of mass media awareness raising (MAR) is $5,650 per DALY, yet, basic awareness raising (BAR) costs $13,713 per DALY.

Screening intervention combinations have ACERs ranging from $4,125 to $10,939 per DALY. The most cost-effective screening intervention in our analysis is triennial fixed mammography screening (age 45-69) in urban areas combined with mobile mammography screening (age 45-69) in non-urban areas, which costs $4,125 per DALY averted.

Figure 2 shows the cost-effectiveness thresholds of three times ($12,204) and one time ($4,608) the Peruvian GDP per capita per DALY averted (dotted lines). This figure and Table 5 also show the expansion path for breast cancer control, i.e. the order in which interventions should be implemented at different levels of resource availability on the basis of incremental cost-effectiveness ratio (ICER). This path shows that triennial fixed mammography screening (age 45-69) in urban areas combined with mobile mammography screening (age 45-69) in non-urban areas is the optimal choice ($4,125 per DALY averted, scenario 67), followed by triennial fixed mammography screening (age 40-69) in urban areas combined with mobile mammography screening (age 40-69) in non-urban (ICER of $5,659 per DALY averted, scenario 65). After that, the next best intervention that follows from this expansion path is biennial mammography screening (40-69 years) with fixed and mobile units (ICER $27,477 per DALY, scenario 59). These screening interventions are all combined with treatment of all stages and standard palliative care (SPC). Eventually, annual fixed and mobile screening combined with extended palliative care and trastuzumab (scenario 94) is the most extensive intervention with an ICER of $87,243 per DALY averted. 

**Table 5:** Recommended interventions according to their incremental cost-effectiveness ratio (ICER), position in expansion path and budget impact.

**Sce-nario number (#)**	**Intervention scenarios**	**Coverage level (%)**	**Patients per year**	Annual treatment costs**	Annual program costs**	Annual training costs**	Annual total costs**	Cost per patient a year**	DALYs averted a year***	DALYs averted per patient a year***	**ACER**	**ICER**
4	Stage I treatment & relapse only	95%	1,602	6,582,278	515,816	29,227	7,127,321	4,449	1,318	0,82	5,406	Dominated
85	Stage I to IV treatment with triennial MIXED screening: URBAN (45-49 CBE) (50-69 MM FIXED) 60%/ RURAL (CBE 45-69) 40%*	95%	4,402	53,035,136	10,396,581	276,684	63,708,401	14,473	14,308	3,25	4,453	Dominated
83	Stage I to IV treatment with triennial MIXED screening: URBAN (40-49 CBE) (50-69 MM FIXED) 60%/ RURAL (CBE 40-69) 40%*	95%	4,402	53,577,050	10,396,581	276,684	64,250,315	14,596	14,959	3,40	4,295	Dominated
89	Stage I to IV treatment with most efficient triennial MIXED: URBAN (40-49 CBE) (50-69 MM FIXED) 60%/ RURAL (CBE 40-69) 40%*+ FNA*	95%	4,402	53,557,982	11,208,251	292,272	65,058,506	14,779	14,959	3,40	4,349	Dominated
90	Stage I to IV treatment with most efficient triennial MIXED: URBAN (40-49 CBE) (50-69 MM FIXED) 60%/ RURAL (CBE 40-69) 40%* + FNA + BPC	95%	4,402	53,539,583	12,511,232	518,783	66,569,598	15,123	14,961	3,40	4,450	Dominated
67	Stage I to IV treatment with triennial mammography screening (45-69 years) FIXED 60%/MOBILE 40%*	95%	4,402	54,944,080	13,423,175	350,727	68,717,982	15,611	16,657	3,78	4,125	4,125
91	Stage I to IV treatment with most efficient triennial FIXED/MOBILE screening strategy (FIXED/MOBILE, 45-69) + BPC	95%	4,402	54,804,394	14,726,156	577,237	70,107,788	15,926	16,658	3,78	4,209	Dominated
65	Stage I to IV treatment with triennial mammography screening (40-69 years) FIXED 60%/MOBILE 40%*	95%	4,402	57,581,446	13,423,175	350,727	71,355,347	16,210	17,123	3,89	4,167	5,659
60	Stage I to IV treatment with biennial mammography screening (40-64 years) FIXED 60%/MOBILE 40%*	95%	4,402	62,065,226	15,710,263	370,211	78,145,701	17,752	17,338	3,94	4,507	Dominated†
59	Stage I to IV treatment with biennial mammography screening (40-69 years) FIXED 60%/MOBILE 40%*	95%	4,402	63,804,007	15,710,263	370,211	79,884,482	18,147	17,433	3,96	4,582	27,477†
55	Stage I to IV treatment with annual mammography screening (45-69 years) FIXED 60%/MOBILE 40%*	95%	4,402	74,070,789	17,997,352	389,696	92,457,837	21,004	17,385	3,95	5,318	Dominated†
53	Stage I to IV treatment with annual mammography screening (40-69 years) FIXED 60%/MOBILE 40%*	95%	4,402	83,070,430	17,997,352	389,696	101,457,478	23,048	17,857	4,06	5,682	Dominated†
94	Stage I to IV treatment with most expensive screening strategy (annual, FIXED60%/MOBILE40%, 40-69 ) + EPC + trastuzumab (all stages)	95%	4,402	103,306,498	19,638,424	625,949	123,570,871	28,072	18,737	4,26	6,595	87,243†

**ICER**: Incremental cost effectiveness ratio, ratio of additional cost per additional life-year saved when next intervention is added to a mix (additional US$ per additional DALY saved). **ACER**: Average cost-effectiveness ratio compared to the do nothing-scenario (US$ per DALY averted). **MIXED** screening: combines both CBE screening and mammography screening elements in the screening program. **URBA**N: program specified for urban population, covers about 60% of the total population. **RURAL**: program specified for rural population, covers about 40% of the total population. CBE: clinical breast examination screening. MM: mammography screening. FIXED: screening program based on fixed mammography units. **MOBILE**: screening program based on mobile screening unit. **FNA**: upfront fine needle aspiration program. **BPC**: basic palliatice care program. **EPC**: extende palliative care program.

* These scenarios include Standard Palliative Care (SPC)

** In 2012 US$ (1 SOL = 0,384 US$)

*** DALYs, disability-adjusted life-years (age weighted, 3% discounted) . DALYs are averted over a 100 year period but attributed to the implementation period of 10 years.

^†^ These interventions have ICERs higher than the 3 times GDP per capita per DALY threshold and can, strictly speaking, not be considered cost-effective.

Note that of the aforementioned interventions, the ICERs of only 2 interventions (scenario 67 and 65) are beneath the proposed cost-effectiveness thresholds. Therefore, strictly interpreted, they are the only candidates for implementation in Peru according to the rules of allocative efficiency. Additionally, other interventions (scenarios #4, #53, #55, #60, #83, #85, #90-#93) are not on the expansion path (i.e. dominated) and should therefore not be considered as well. However, as these small differences in ICERs are likely not relevant at the policy level, we nevertheless consider interventions on - and close to - the expansion path as potential candidates for implementation in Peru (Table 5). 

### Sensitivity Analysis

Sensitivity analysis showed that our model is most sensitive to alternative assumptions on screening attendance and the sensitivity of screening devices. Varying the case fatality rates and current stage distribution also impacts our results, whereas alternative assumptions on unit costs for FNA or mammography, transportation multipliers or DW’s have less impact (Table S3). Lowering screening attendance from 72% to 54% (-25%) would increase the ACERs with about 26%, while lower test sensitivities of CBE and mammography screens (-25%) would increase the ACERs with about 24%. If higher case-fatality rates were assumed (+25%), representing poorer survival, the ACERs of the interventions in table S3 would increase about 22%. Lower case-fatality rates would result in a 15% decrease of these ACERs. Increased intervention costs due to respectively higher unit costs of FNA, mammograms and transportation multipliers (+25%) increase the ACERs between 0% and 9%.

## Discussion

We have quantified the health effects, costs, and cost-effectiveness of a broad range of interventions for breast cancer control in Peru. The results were obtained by means of a dynamic population model, using consistent demographic and epidemiological data of the populations, allowing general comparisons of the costs and effects of the interventions studied. 

Our results provide important information on strategies for breast cancer control in Peru and suggest that the current situation in Peru could be improved through implementation of triennial or biennial mammography screening strategies, combined with treatment of all strategies and standard palliative care. These strategies seem the most cost-effective in Peru, and costs between $68 and $80 million per year. Probably also cost-effective, but less expensive, are triennial screening strategies through combining mammography and CBE screening. These strategies, combined with or without basic palliative care or upfront FNA, cost between $64 and $66 million per year. Annual screening strategies come with higher cost to the healthcare system and with relatively lower effects compared to tri- or biennial screening, and are therefore not recommended from an economic perspective.

Of the abovementioned interventions, only triennial mammography screening strategies can be labeled cost-effective (scenario 67 and 65). However, considering the uncertainty on the effectiveness of these interventions, and considering the inappropriateness to use this threshold as the sole criterion for choosing interventions at the policy level, we suggest considering all the interventions near the expansion path for planning (long term) strategies (Table 5). Besides the efficiency aspects of the studied interventions, we believe the choice of intervention should also relate to other aspects of the health system such as budget impact, equity and feasibility. These aspects are discussed below.

First, compared to mammography screening, CBE screening with upfront FNA implies a simpler and technically less demanding approach at the primary healthcare level. Although the total costs of adding the upfront FNA component are slightly higher (about $240,000 per year), patient costs can be slightly reduced due to the lower costs of the FNA strategy ($3.48 saved per diagnosis) and the implementation of this intervention has been demonstrated in a very rural area [25]. This intervention, which includes an awareness raising of signs and symptoms component, could be recommended above usual CBE screening strategies in Peru for feasibility reasons, especially in rural areas. 

Second, although treatment interventions are - on themselves - not economically attractive, treatment is an integral component of the continuum of care and essential to be scaled up if any screening intervention is implemented. Only 60% of the Peruvian population is currently insured, creating high barriers to accessing care for many Peruvians. Besides treatment interventions, awareness raising of signs and symptoms (particularly in areas where breast cancer is diagnosed in late stages) is imperative for early detection [50]. Also, if any form of early detection or screening is implemented, patients need to be referred through to a comprehensive system with low social and financial barriers. This could partly be managed by reimbursing patients and their families for travel and accommodation. Efforts to reach universal coverage should therefore continue and a gradual increase in coverage of current treatment services, along with improvements of referral systems should first - or simultaneously - be established in Peru. 

Third, stage IV treatment only (including standard palliative care (SPC)) is the least economically attractive intervention (ACER of $48,576 per DALY), and generally palliative care cannot be recommended from an economic perspective. If management of stage IV patients entails home based visits (BPC), patient costs slightly decrease due to a reduction of hospitalization days. However, the extra training and program cost for organizing this palliative service model outweigh these savings and BPC is not cost-effective either. Nevertheless, this intervention costs only slightly more than the current SPC ($1.2 million more) and allows patients to decease at home, where family and friends are able to support and spend their last moments with the patient. For this reason, and regarding the many patients in advanced stages currently, it could be meaningful to provide basic palliative care in Peru.

Fourth, the addition of trastuzumab to all eligible patients (about 15% of all patients), is less economically attractive than treatment of all stages (ACER of $10,620 vs. ACER of $8,605 per DALY). Moreover, it comes with an additional cost of over $25 million ($83.8 million in total) - almost 45% higher than the budget for treatment of all stages. If trastuzumab is given only to eligible patients in stage I and II, this ACER is lower ($9,247 per DALY) and the additional costs are about 12% higher ($65.5 million in total). This intervention should therefore be preferred if trastuzumab is added as a therapeutic option for breast cancer control. The addition of trastuzumab to all eligible patients is not recommended for Peru.

Fifth, breast cancer screening highly depends on the availability of human resources, facilities and devices for proper diagnosis and treatment. It is necessary to secure adequate infrastructure, equipment and human resources before any screening activities can commence. In addition, as the current health system in Peru is fragmented and in a decentralization process, it seems very difficult to achieve nationwide, organized screening. If screening is provided by competitive public and private actors, all with their own target populations in the same areas, we recommend either law enforcement and strong leadership to negotiate a plan with all these actors, or installing a separate public operation that could provide the entire screening programme. Attendance rates are perhaps equally important for both the success of screening and the equitable distribution of its health outcomes. In this, appropriate education and information is essential and although most screening studies show positive results on stage distribution in developing countries [51-53], these interventions can easily fail when education and information are neglected. Screening or early detection communications strategies should also include clear messages on the benefits and harms of the different early detection modalities [50]. Our sensitivity analysis furthermore showed that if attendance rates reduce from 72% to 54%, the ACERs of screening interventions increase with 26%. We therefore recommend a thorough evaluation of Peru’s current screening activities, so these barriers become more transparent and future screening programs can better guarantee adequate attendance and equal access.

Sixth, mobile screening units are generally more accessible in non-urban areas as opposed to fixed mammography units and therefore more effective. Mobile screening could also lead to a more equal distribution of health outcomes and could therefore be considered if screening is implemented. However, the costs for reaching out to the non-urban areas (30%-40% of total population) by mobile units are high as the cost of each screen increases with at least 20%. A combination of CBE screening and mammography screening (mixed screening) seems a cost-effective alternative with lower budget impact, and less complex to implement in non-urban areas compared to mobile mammography screening. Hence, we generally recommend Peru to consider a mixed screening strategy (CBE screening below 50 and mammography screening in women older than 50 for urban areas, and CBE screening in all ages for non-urban areas) for feasibility and budgetary reasons.

Seventh, and in general, the current budget for controlling priority cancers in Peru (colo-rectal, stomach, cervical, breast, prostate, lymphomas, leukemia) has been increased to over $25 million for 2012 [11]. Despite this impetus, the full implementation of the broad range of breast cancer interventions already requires more budget. Treatment of all patients with breast cancer would costs around $58 million per year, and screening will at least cost another $5 million per year. Moreover, the budget for (breast) cancer control also faces competition with other healthcare interventions. International literature suggests that interventions for communicable diseases and preventive interventions for non-communicable diseases are economically more attractive compared to breast cancer interventions [20,54,55]. With regards to the economic attractiveness of screening interventions for other non-communicable diseases, breast cancer screening seems to compare worse to cervical screening but better compared to colorectal screening [16]. Yet, these international estimates should be carefully interpreted for national level decision making. Given these budgetary constraints, the MoH in Peru could decide to implement less expensive interventions such as CBE screening, mass-media awareness raising, or treatment only. This would however introduce an inefficient use of resources and instead we suggest to gradually expand the recommended screening interventions, starting at lower -more affordable- coverage levels. The MoH in Peru could for example first increase treatment coverage and select an urban area to demonstrate triennial CBE and mammography screening in currently targeted women (45-64 years). In a non-urban area, awareness raising and CBE screening could first be initiated and combined with upfront FNA. Once a reasonable increase in coverage is reached, the program could expand to screening women 45 to 69 years, or 40-69 years old, possibly by mobile units providing both mammography and CBE. These mobile units could be shared for the screening and early detection activities of the other priority diseases. The gradual expansion will give extra time to train the required human resources and to negotiate more budget for infrastructure and equipment. 

Our study has a number of limitations. First, a national cancer registry in Peru is not yet available and local data on breast cancer epidemiology and patient resource patterns were derived from different sources. Breast cancer treatment practices probably differ between the many public and private institutions. Since our data was mostly based on composite hospital data from the urban, public sector, our results may not be representative for the whole country. These limitations indicate the need to start a national cancer registry in Peru. Second, evidence on the effectiveness of awareness raising, CBE and mammography screening in Peru and many other countries is absent. To arrive at Peruvian estimates we used a model approach that has previously been applied in a range of other studies and was also considered credible by the expert panel in our study. Also, our sensitivity analysis shows that using alternative assumptions on case fatality rates, attendance rates or the sensitivity of screening devices lead to significant differences in cost-effectiveness. A combined effect of these factors could change the cost-effectiveness of the interventions under study further. However, as these factors have a similar impact on all interventions under study, it is unlikely that this combined effect would change our study conclusions. Despite these limitations, the results of our model show similarities with results from other models [52,56,57]. Third, as information on the patient resource patterns of the upfront FNA strategy was limited in Peru, we assumed similar final outcomes for both CBE screening with upfront FNA and the usual CBE screening strategy (i.e. the number benign or malignant outcomes in both arms in Figure S1). However, FNA could also cause structural distortions that may render further imaging accuracy. Fourth, in the absence of reliable data and following the health care perspective of the Peruvian MoH, we did not include travel costs or productivity losses of patients seeking or undergoing care. Including these cost would have probably led to increased cost generally, and particularly for women with advanced stage breast cancer [58,59]. WHO-CHOICE analyses aim to provide broad indications of cost-effectiveness on a range of interventions to inform general policy discussions rather than to deliver very precise estimates on a specific intervention and the above limitations are a manifestation of this.

## Conclusions

In summary, taking in consideration cost-effectiveness and other factors, our analysis suggests that CBE screening with upfront FNA in non-urban settings (age 40-69), combined with both CBE and fixed mammography screening in urban settings (age 40-69), could be a preliminary, cost-effective and feasible option for Peru. A combination of fixed and mobile mammography screening, due to its high budget impact and the challenging implementation characteristics, should perhaps be preferred on the long term when the economic and health system conditions improve. However, whichever screening modality is used, awareness raising of signs and symptoms, cancer diagnosis, cancer treatment and basic palliative care services should be improved simultaneously and barriers to early detection and breast cancer care along the continuum should also be explored and dissolved. As population based screening programs are very complex and resource intensive, particularly mammography screening, we suggest Peru to focus initially on triennial screening in women currently targeted (age 45-64) in urban and non-urban demonstration areas and gradually expand to the proposed program. Annual screening strategies, late stage treatment and trastuzumab therapy are generally not economically attractive.

## Supporting Information

Figure S1 **Comparison of usual CBE screening strategy and CBE screening with upfront FNA, and level of execution.** *CBE* screening with upfront FNA (fine needle aspiration): after a positive CBE screen (about 4% of the CBE screened population) women receive FNA. Depending on the FNA test results, mammography (MM) or core needle biopsy (CNB) is performed as part of the triple test (physical examination, mammography, needle biopsy) for final breast cancer diagnosis.
(TIF)

Table S1 **Example of micro costing study results (i.e. core biopsy), and their modifications in Peru.**
(DOCX)

Table S2 **Costs (US$), effects and cost-effectiveness of all analyzed breast cancer control interventions in Peru.**
(DOCX)

Table S3 **Results of sensitivity analysis on average cost-effectiveness ratios (ACERs) of recommended interventions.**
(DOCX)
