# Supplementary figures and images for: Cost-Effectiveness Analysis of Breast Cancer Control Interventions in Peru
Source: PLoS One. 2013 Dec 10;8(12):e82575. doi: 10.1371/journal.pone.0082575 (PMC3859673; doi:10.1371/journal.pone.0082575)

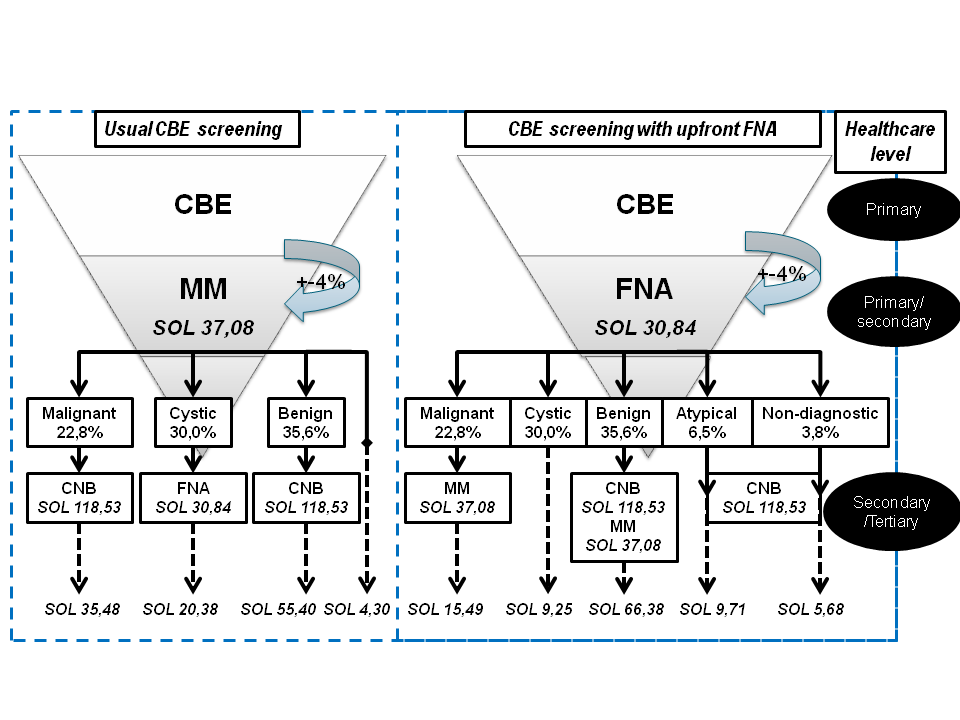

Supplement: Figure S1 — Comparison of usual CBE screening strategy and CBE screening with upfront FNA, and level of execution. CBE screening with upfront FNA (fine needle aspiration): after a positive CBE screen (about 4% of the CBE screened population) women receive FNA. Depending on the FNA test results, mammography (MM) or core needle biopsy (CNB) is performed as part of the triple test (physical examination, mammography, needle biopsy) for final breast cancer diagnosis. (TIF) [file pone.0082575.s001.tif]
