# Supplementary material for: Cost-Effectiveness Analysis of Breast Cancer Control Interventions in Peru
Source: PLoS One. 2013 Dec 10;8(12):e82575. doi: 10.1371/journal.pone.0082575 (PMC3859673; doi:10.1371/journal.pone.0082575)
Supplement: Table S1 — Example of micro costing study results (i.e. core biopsy), and their modifications in Peru. (DOCX) [file pone.0082575.s002.docx]

**Table S1.** **Example of micro costing study results and their modifications in Peru.**

| **CORE BIOPSY MICRO COSTING** | | | | | | | **Multiplier drugs/goods*** |
| --- | --- | --- | --- | --- | --- | --- | --- |
| **HUMAN RESOURCES** |  | **Time (min)** |  | **HR wage** | **HR per min.** | **Cost per procedure** |  |
| Medical doctor |  | 10 |  | 4,500.00 | 0.50000 | 5.00000 |  |
| Nursing assistent |  | 10 |  | 1,800.00 | 0.20000 | 2.00000 |  |
| Total |  |  |  |  |  | **7.00000** |  |
| **REUSABLE ITEMS** |  | **Time (min)** | **Buying price** | **Useful Life** | **Depreciation per min.** | **Cost per procedure** |  |
| Trolley |  | 10 | 500.00 | 10 | 0.00010 | 0.00096 |  |
| Metal stretcher |  | 10 | 968.00 | 10 | 0.00019 | 0.00187 |  |
| Total |  |  |  |  |  | **0.00283** |  |
| **DISPOSABLE ITEMS** | **Presentation** | **Buying price** | **Unit definition** | **quantity used** | **Price per unit** | **Cost per procedure** |  |
| 1. Activity: Skin Cleaning |  |  |  |  |  |  |  |
| Gauze (x 2 und) |  |  | Pack | 3 | 0.45442 | 1.36325 |  |
| Alcohol |  |  | Ml | 1 | 0.02079 | 0.02079 |  |
| Formaldehyde |  |  | Ml | 5 | 0.02140 | 0.10700 |  |
| Plaster |  |  | Cm | 20 | 0.01067 | 0.21333 |  |
| 2. Activity: Procedure |  |  |  |  |  |  |  |
| 5cc disposable syringe. C / A 21x1 1/2" |  |  | Piece | 1 | 0.13000 | 0.13000 |  |
| Disposable needle 25X5/8X100 |  |  | Piece | 1 | 0.06800 | 0.06800 |  |
| 21x1 disposable needle 1/2X100 |  |  | Piece | 1 | 0.06800 | 0.06800 |  |
| Biopsy Needle 14 x 10 |  |  | Piece | 1 | 90.25000 | 90.25000 |  |
| 25.10cm x 24.5cm Paper Towel x 175 sheets | 175 | 6.46 | Sheet | 4 | 0.03691 | 0.14766 |  |
| Germicidal Soap Liquid x 800ml | 800 | 11.00 | Ml | 6 | 0.01375 | 0.08250 |  |
| Total |  |  |  |  |  | **92.45053** | **106.32366** |
| **FACILITIES**** | **Time (min)** | **Size** | **Useful Life** | **Replacement costs per m2** | **cost per m2** | **Cost per procedure** |  |
| Examination / procedure room | 12.5 | 12.5 | 15 | 3120 | 0.17265 | 0.44962 |  |
| **DRUGS & MEDICATION** | **Presentation** | **Buying price** | **Unit definition** | **quantity used** | **Price per unit** | **Costs per procedure** |  |
| Xilocaine 2% |  |  | Fco | 1 | 3.50000 | 3.50000 |  |
|  |  |  |  |  |  | **3.50000** | **5.20205** |
|  |  |  |  |  |  |  |  |
| **TOTAL COSTS** |  |  |  |  |  | **102.95** | **118.52854** |

**This procedure does not include specimen examination.**

***** Drug and goods multipliers are derived from WHO-CHOICE database and are used to correct for cost of shipping and transportation. Multipliers are 1,4863 for drugs, and 1,1501 for goods.

****** Details on facilities also derived from CHOICE database. Annualization (r=3%) is: A= ((1+r)^useful life-1)/(r*(1+r)^useful life). Maintenance costs for facility is 7%, capacity utilization is 240 working days for 8 hours a day [[20](#_ENREF_20)].
