# Supplementary material for: Cost-Effectiveness Analysis of Breast Cancer Control Interventions in Peru
Source: PLoS One. 2013 Dec 10;8(12):e82575. doi: 10.1371/journal.pone.0082575 (PMC3859673; doi:10.1371/journal.pone.0082575)
Supplement: Table S2 — Costs (US$), effects and cost-effectiveness of all analyzed breast cancer control interventions in Peru. (DOCX) [file pone.0082575.s003.docx]

**Table S2. Costs (US$), effects and cost-effectiveness of breast cancer control in Peru.**

| **#** | **Intervention scenarios** | **Coverage level (%)** | **Patients per year** | **Annual treatment costs**** | **Annual program costs**** | **Annual training costs**** | **Annual total costs**** | **DALYs averted a year***** | **ACER** | **ICER** |
| --- | --- | --- | --- | --- | --- | --- | --- | --- | --- | --- |
| 1 | Stage I to IV treatment combined (60% coverage)* | 60% | 3,340 | 34,311,601 | 1,515,565 | 128,543 | 35,955,708 | 4,274 | 8,412 | Dominated |
| 2 | Stage I to IV treatment combined (80% coverage)* | 80% | 4,453 | 44,627,285 | 2,180,260 | 132,064 | 46,939,609 | 5,699 | 8,237 | Dominated |
| 3 | Current country specific situation (50%), limited awareness, opportunistic screening (15%), counseling 18-64 (30%)* | 50% | 2,783 | 38,036,892 | 5,947,640 | 154,144 | 44,138,676 | 5,238 | 8,426 | Dominated |
| 4 | Stage I treatment & relapse only | 95% | 372 | 6,582,278 | 515,816 | 29,227 | 7,127,321 | 1,318 | 5,406 | Dominated |
| 5 | Stage II treatment & relapse only* | 95% | 1,927 | 22,430,570 | 564,700 | 29,227 | 23,024,497 | 2,900 | 7,940 | Dominated |
| 6 | Stage III treatment & relapse only* | 95% | 2,299 | 22,208,226 | 564,700 | 29,227 | 22,802,153 | 2,245 | 10,157 | Dominated |
| 7 | Stage IV treatment only* | 95% | 690 | 20,560,745 | 1,283,145 | 48,712 | 21,892,603 | 451 | 48,576 | Dominated |
| 8 | Stage I to IV treatment combined (95%)* | 95% | 5,288 | 54,815,497 | 3,279,962 | 136,394 | 58,231,853 | 6,767 | 8,605 | Dominated |
| 9 | Stage I to IV treatment combined (95%) + I-IV trastuzumab* | 95% | 5,288 | 80,369,660 | 3,330,701 | 146,136 | 83,846,497 | 7,895 | 10,620 | Dominated |
| 10 | Stage I to IV treatment combined (95%) + stage I&II trastuzumab* | 95% | 5,288 | 61,993,558 | 3,330,701 | 146,136 | 65,470,395 | 7,080 | 9,247 | Dominated |
| 11 | BPC: Palliative care basic + treatment stage I to III (voluntary home based visits, morphine, laxatives and radiotherapy) + end of life hospitalization | 95% | 5,288 | 54,505,724 | 4,582,943 | 362,905 | 59,451,571 | 6,769 | 8,782 | Dominated |
| 12 | EPC: Palliative care extended + treatment stage I to III (trained staff home based visits, morphine, anti depressives, anti emetics, radiotherapy, biphosphonates and laxatives) + end of life hospitalization | 95% | 5,288 | 54,571,376 | 4,870,295 | 362,905 | 59,804,575 | 6,771 | 8,832 | Dominated |
| 13 | BAR: Stage I to IV treatment with basic awareness outreach program* | 95% | 5,288 | 62,640,679 | 9,954,187 | 165,621 | 72,760,486 | 5,306 | 13,713 | Dominated |
| 14 | MAR: Stage I to IV treatment with basic awareness outreach program + mass media campaign* | 95% | 5,288 | 52,877,241 | 15,400,957 | 165,621 | 68,443,818 | 12,115 | 5,650 | Dominated |
| 15 | Stage I to IV treatment with annual CBE screening (40-69 years)* | 95% | 5,288 | 63,204,934 | 14,094,460 | 292,272 | 77,591,666 | 14,085 | 5,509 | Dominated |
| 16 | Stage I to IV treatment with annual CBE screening (40-64 years)* | 95% | 5,288 | 62,930,253 | 14,094,460 | 292,272 | 77,316,985 | 12,788 | 6,046 | Dominated |
| 17 | Stage I to IV treatment with annual CBE screening (45-69 years)* | 95% | 5,288 | 60,166,289 | 14,094,460 | 292,272 | 74,553,021 | 13,137 | 5,675 | Dominated |
| 18 | Stage I to IV treatment with annual CBE screening (45-64 years)* | 95% | 5,288 | 60,316,751 | 14,094,460 | 292,272 | 74,703,483 | 11,673 | 6,400 | Dominated |
| 19 | Stage I to IV treatment with annual CBE screening (50-69 years)* | 95% | 5,288 | 60,683,256 | 14,094,460 | 292,272 | 75,069,988 | 9,633 | 7,793 | Dominated |
| 20 | Stage I to IV treatment with annual CBE screening (50-64 years)* | 95% | 5,288 | 61,324,047 | 14,094,460 | 292,272 | 75,710,779 | 7,659 | 9,886 | Dominated |
| 21 | Stage I to IV treatment with biennial CBE screening (40-69 years)* | 95% | 5,288 | 56,162,452 | 12,182,647 | 272,787 | 68,617,887 | 13,025 | 5,268 | Dominated |
| 22 | Stage I to IV treatment with biennial CBE screening (40-64 years)* | 95% | 5,288 | 56,927,228 | 12,182,647 | 272,787 | 69,382,662 | 11,680 | 5,940 | Dominated |
| 23 | Stage I to IV treatment with biennial CBE screening (45-69 years)* | 95% | 5,288 | 55,287,877 | 12,182,647 | 272,787 | 67,743,312 | 12,086 | 5,605 | Dominated |
| 24 | Stage I to IV treatment with biennial CBE screening (45-64 years)* | 95% | 5,288 | 56,194,645 | 12,182,647 | 272,787 | 68,650,080 | 10,514 | 6,530 | Dominated |
| 25 | Stage I to IV treatment with biennial CBE screening (50-69 years)* | 95% | 5,288 | 57,411,326 | 12,182,647 | 272,787 | 69,866,761 | 8,527 | 8,193 | Dominated |
| 26 | Stage I to IV treatment with biennial CBE screening (50-64 years)* | 95% | 5,288 | 58,685,453 | 12,182,647 | 272,787 | 71,140,888 | 6,629 | 10,731 | Dominated |
| 27 | Stage I to IV treatment with triennial CBE screening (40-69 years)* | 95% | 5,288 | 54,283,411 | 10,396,581 | 253,302 | 64,933,295 | 12,328 | 5,267 | Dominated |
| 28 | Stage I to IV treatment with triennial CBE screening (40-64 years)* | 95% | 5,288 | 55,308,356 | 10,396,581 | 253,302 | 65,958,240 | 10,964 | 6,016 | Dominated |
| 29 | Stage I to IV treatment with triennial CBE screening (45-69 years)* | 95% | 5,288 | 54,035,784 | 10,396,581 | 253,302 | 64,685,668 | 11,398 | 5,675 | Dominated |
| 30 | Stage I to IV treatment with triennial CBE screening (45-64 years)* | **95%** | 5,288 | 55,183,240 | 10,396,581 | 253,302 | 65,833,123 | 9,910 | 6,643 | Dominated |
| 31 | Stage I to IV treatment with triennial CBE screening (50-69 years)* | 95% | 5,288 | 56,636,210 | 10,396,581 | 253,302 | 67,286,094 | 7,993 | 8,418 | Dominated |
| 32 | Stage I to IV treatment with triennial CBE screening (50-64 years)* | 95% | 5,288 | 56,636,210 | 10,396,581 | 253,302 | 67,286,094 | 6,173 | 10,900 | Dominated |
| 33 | Stage I to IV treatment with most efficient biennial CBE screening strategy (45-69) + FNA* | **95%** | 5,288 | 55,264,759 | 12,425,756 | 292,272 | 67,982,787 | 12,086 | 5,625 | Dominated |
| 34 | Stage I to IV treatment with most efficient triennial CBE screening strategy (40-69) +FNA* | 95% | 5,288 | 54,260,293 | 10,639,690 | 272,787 | 65,172,770 | 12,328 | 5,287 | Dominated |
| 35 | Stage I to IV treatment with annual mammography screening (40-69 years) FIXED 60%* | 95%/60% | 5,288 | 79,874,043 | 14,094,460 | 311,757 | 94,280,260 | 12,876 | 7,322 | Dominated |
| 36 | Stage I to IV treatment with annual mammography screening (40-64 years) FIXED 60%* | 95%/60% | 5,288 | 77,919,860 | 14,094,460 | 311,757 | 92,326,077 | 12,073 | 7,648 | Dominated |
| 37 | Stage I to IV treatment with annual mammography screening (45-69 years) FIXED 60%* | 95%/60% | 5,288 | 72,828,089 | 14,094,460 | 311,757 | 87,234,306 | 12,580 | 6,934 | Dominated |
| 38 | Stage I to IV treatment with annual mammography screening (45-64 years) FIXED 60%* | 95%/60% | 5,288 | 70,995,163 | 14,094,460 | 311,757 | 85,401,380 | 11,665 | 7,321 | Dominated |
| 39 | Stage I to IV treatment with annual mammography screening (50-69 years) FIXED 60%* | 95%/60% | 5,288 | 67,910,345 | 14,094,460 | 311,757 | 82,316,562 | 11,196 | 7,352 | Dominated |
| 40 | Stage I to IV treatment with annual mammography screening (50-64 years) FIXED 60%* | 95%/60% | 5,288 | 66,641,117 | 14,094,460 | 311,757 | 81,047,334 | 9,756 | 8,308 | Dominated |
| 41 | Stage I to IV treatment with biennial mammography screening (40-69 years) FIXED 60%* | 95%/60% | 5,288 | 64,909,615 | 12,245,521 | 302,014 | 77,457,150 | 12,610 | 6,142 | Dominated |
| 42 | Stage I to IV treatment with biennial mammography screening (40-64 years) FIXED 60%* | 95%/60% | 5,288 | 64,456,078 | 12,245,521 | 302,014 | 77,003,613 | 11,741 | 6,558 | Dominated |
| 43 | Stage I to IV treatment with biennial mammography screening (45-69 years) FIXED 60%* | 95%/60% | 5,288 | 61,548,429 | 12,245,521 | 302,014 | 74,095,964 | 12,318 | 6,015 | Dominated |
| 44 | Stage I to IV treatment with biennial mammography screening (45-64 years) FIXED 60%* | 95%/60% | 5,288 | 61,210,649 | 12,245,521 | 302,014 | 73,758,184 | 11,343 | 6,503 | Dominated |
| 45 | Stage I to IV treatment with biennial mammography screening (50-69 years) FIXED 60%* | 95%/60% | 5,288 | 59,878,901 | 12,245,521 | 302,014 | 72,426,436 | 10,918 | 6,634 | Dominated |
| 46 | Stage I to IV treatment with biennial mammography screening (50-64 years) FIXED 60%* | 95%/60% | 5,288 | 60,084,504 | 12,245,521 | 302,014 | 72,632,039 | 9,432 | 7,700 | Dominated |
| 47 | Stage I to IV treatment with triennial mammography screening (40-69 years) FIXED 60%* | 95%/60% | 5,288 | 60,041,220 | 10,396,581 | 292,272 | 70,730,073 | 12,415 | 5,697 | Dominated |
| 48 | Stage I to IV treatment with triennial mammography screening (40-64 years) FIXED 60%* | 95%/60% | 5,288 | 60,110,494 | 10,396,581 | 292,272 | 70,799,347 | 11,503 | 6,155 | Dominated |
| 49 | Stage I to IV treatment with triennial mammography screening (45-69 years) FIXED 60%* | 95%/60% | 5,288 | 57,910,077 | 10,396,581 | 292,272 | 68,598,931 | 12,122 | 5,659 | Dominated |
| 50 | Stage I to IV treatment with triennial mammography screening (45-64 years) FIXED 60%* | 95%/60% | 5,288 | 58,092,041 | 10,396,581 | 292,272 | 68,780,894 | 11,106 | 6,193 | Dominated |
| 51 | Stage I to IV treatment with triennial mammography screening (50-69 years) FIXED 60%* | 95%/60% | 5,288 | 57,331,690 | 10,396,581 | 292,272 | 68,020,543 | 10,708 | 6,352 | Dominated |
| 52 | Stage I to IV treatment with triennial mammography screening (50-64 years) FIXED 60%* | 95%/60% | 5,288 | 58,040,879 | 10,396,581 | 292,272 | 68,729,733 | 9,194 | 7,475 | Dominated |
| 53 | Stage I to IV treatment with annual mammography screening (40-69 years) FIXED 60%/MOBILE 40%* | 95% | 5,288 | 83,070,430 | 17,997,352 | 389,696 | 101,457,478 | 17,857 | 5,682 | Dominated |
| 54 | Stage I to IV treatment with annual mammography screening (40-64 years) FIXED 60%/MOBILE 40%* | 95% | 5,288 | 80,876,649 | 17,997,352 | 389,696 | 99,263,697 | 16,578 | 5,988 | Dominated |
| 55 | Stage I to IV treatment with annual mammography screening (45-69 years) FIXED 60%/MOBILE 40%* | 95% | 5,288 | 74,070,789 | 17,997,352 | 389,696 | 92,457,837 | 17,385 | 5,318 | Dominated |
| 56 | Stage I to IV treatment with annual mammography screening (45-64 years) FIXED 60%/MOBILE 40%* | 95% | 5,288 | 72,079,104 | 17,997,352 | 389,696 | 90,466,152 | 15,928 | 5,680 | Dominated |
| 57 | Stage I to IV treatment with annual mammography screening (50-69 years) FIXED 60%/MOBILE 40%* | 95% | 5,288 | 68,278,794 | 17,997,352 | 389,696 | 86,665,842 | 15,178 | 5,710 | Dominated |
| 58 | Stage I to IV treatment with annual mammography screening (50-64 years) FIXED 60%/MOBILE 40%* | 95% | 5,288 | 67,226,604 | 17,997,352 | 389,696 | 85,613,652 | 12,862 | 6,656 | Dominated |
| 59 | Stage I to IV treatment with biennial mammography screening (40-69 years) FIXED 60%/MOBILE 40%* | 95% | 5,288 | 63,804,007 | 15,710,263 | 370,211 | 79,884,482 | 17,433 | 4,582 | 27,477 |
| 60 | Stage I to IV treatment with biennial mammography screening (40-64 years) FIXED 60%/MOBILE 40%* | 95% | 5,288 | 62,065,226 | 15,710,263 | 370,211 | 78,145,701 | 17,338 | 4,507 | Dominated |
| 61 | Stage I to IV treatment with biennial mammography screening (45-69 years) FIXED 60%/MOBILE 40%* | 95% | 5,288 | 59,573,839 | 15,710,263 | 370,211 | 75,654,314 | 16,969 | 4,458 | Dominated |
| 62 | Stage I to IV treatment with biennial mammography screening (45-64 years) FIXED 60%/MOBILE 40%* | 95% | 5,288 | 59,542,467 | 15,710,263 | 370,211 | 75,622,942 | 15,412 | 4,907 | Dominated |
| 63 | Stage I to IV treatment with biennial mammography screening (50-69 years) FIXED 60%/MOBILE 40%* | 95% | 5,288 | 57,993,414 | 15,710,263 | 370,211 | 74,073,889 | 14,732 | 5,028 | Dominated |
| 64 | Stage I to IV treatment with biennial mammography screening (50-64 years) FIXED 60%/MOBILE 40%* | 95% | 5,288 | 58,867,681 | 15,710,263 | 370,211 | 74,948,155 | 12,339 | 6,074 | Dominated |
| 65 | Stage I to IV treatment with triennial mammography screening (40-69 years) FIXED 60%/MOBILE 40%* | 95% | 5,288 | 57,581,446 | 13,423,175 | 350,727 | 71,355,347 | 17,123 | 4,167 | 5,659 |
| 66 | Stage I to IV treatment with triennial mammography screening (40-64 years) FIXED 60%/MOBILE 40%* | 95% | 5,288 | 57,581,446 | 13,423,175 | 350,727 | 71,355,347 | 15,668 | 4,554 | Dominated |
| 67 | Stage I to IV treatment with triennial mammography screening (45-69 years) FIXED 60%/MOBILE 40%* | 95% | 5,288 | 54,944,080 | 13,423,175 | 350,727 | 68,717,982 | 16,657 | 4,125 | 4,125 |
| 68 | Stage I to IV treatment with triennial mammography screening (45-64 years) FIXED 60%/MOBILE 40%* | 95% | 5,288 | 55,601,749 | 13,423,175 | 350,727 | 69,375,651 | 15,034 | 4,615 | Dominated |
| 69 | Stage I to IV treatment with triennial mammography screening (50-69 years) FIXED 60%/MOBILE 40%* | 95% | 5,288 | 54,781,516 | 13,423,175 | 350,727 | 68,555,417 | 14,394 | 4,763 | Dominated |
| 70 | Stage I to IV treatment with triennial mammography screening (50-64 years) FIXED 60%/MOBILE 40%* | 95% | 5,288 | 56,317,895 | 13,423,175 | 350,727 | 70,091,797 | 11,954 | 5,864 | Dominated |
| 71 | Stage I to IV treatment with annual MIXED screening: URBAN (40-49 CBE) (50-69 MM FIXED) 60%/ RURAL (CBE 40-69) 40%* | 95% | 5,288 | 67,389,804 | 14,094,460 | 303,963 | 81,788,227 | 16,149 | 5,065 | Dominated |
| 72 | Stage I to IV treatment with annual MIXED screening: URBAN (40-49 CBE) (50-64 MM FIXED) 60%/ RURAL (CBE 40-64) 40%* | 95% | 5,288 | 66,314,284 | 14,094,460 | 303,963 | 80,712,707 | 14,782 | 5,460 | Dominated |
| 73 | Stage I to IV treatment with annual MIXED screening: URBAN (45-49 CBE) (50-69 MM FIXED) 60%/ RURAL (CBE 45-69) 40%* | 95% | 5,288 | 64,292,103 | 14,094,460 | 303,963 | 78,690,526 | 15,481 | 5,083 | Dominated |
| 74 | Stage I to IV treatment with annual MIXED screening: URBAN (45-49 CBE) (50-64 MM FIXED) 60%/ RURAL (CBE 45-64) 40%* | 95% | 5,288 | 63,412,414 | 14,094,460 | 303,963 | 77,810,837 | 13,934 | 5,584 | Dominated |
| 75 | Stage I to IV treatment with annual MIXED screening: URBAN (50-69 MM FIXED) 60%/ RURAL (CBE 50-69) 40%* | 95% | 5,288 | 63,739,722 | 14,094,460 | 303,963 | 78,138,145 | 12,997 | 6,012 | Dominated |
| 76 | Stage I to IV treatment with annual MIXED screening: URBAN (50-64 MM FIXED) 60%/ RURAL (CBE 50-64) 40%* | 95% | 4,402 | 63,523,825 | 14,094,460 | 303,963 | 77,922,248 | 10,814 | 7,205 | Dominated |
| 77 | Stage I to IV treatment with biennial MIXED screening: URBAN (40-49 CBE) (50-69 MM FIXED) 60%/ RURAL (CBE 40-69) 40%* | 95% | 4,402 | 56,808,769 | 12,220,371 | 290,324 | 69,319,464 | 15,443 | 4,489 | Dominated |
| 78 | Stage I to IV treatment with biennial MIXED screening: URBAN (40-49 CBE) (50-64 MM FIXED) 60%/ RURAL (CBE 40-64) 40%* | 95% | 4,402 | 57,161,621 | 12,220,371 | 290,324 | 69,672,316 | 13,982 | 4,983 | Dominated |
| 79 | Stage I to IV treatment with biennial MIXED screening: URBAN (45-49 CBE) (50-69 MM FIXED) 60%/ RURAL (CBE 45-69) 40%* | 95% | 4,402 | 55,631,048 | 12,220,371 | 290,324 | 68,141,743 | 14,785 | 4,609 | Dominated |
| 80 | Stage I to IV treatment with biennial MIXED screening: URBAN (45-49 CBE) (50-64 MM FIXED) 60%/ RURAL (CBE 45-64) 40%* | 95% | 4,402 | 56,159,681 | 12,220,371 | 290,324 | 68,670,376 | 13,160 | 5,218 | Dominated |
| 81 | Stage I to IV treatment with biennial MIXED screening: URBAN (50-69 MM FIXED) 60%/ RURAL (CBE 50-69) 40%* | 95% | 4,402 | 56,654,872 | 14,299,217 | 331,242 | 71,285,331 | 12,395 | 5,751 | Dominated |
| 82 | Stage I to IV treatment with biennial MIXED screening: URBAN (50-64 MM FIXED) 60%/ RURAL (CBE 50-64) 40%* | 95% | 4,402 | 57,837,362 | 12,220,371 | 290,324 | 70,348,057 | 10,138 | 6,939 | Dominated |
| 83 | Stage I to IV treatment with triennial MIXED screening: URBAN (40-49 CBE) (50-69 MM FIXED) 60%/ RURAL (CBE 40-69) 40%* | 95% | 4,402 | 53,577,050 | 10,396,581 | 276,684 | 64,250,315 | 14,959 | 4,295 | Dominated |
| 84 | Stage I to IV treatment with triennial MIXED screening: URBAN (40-49 CBE) (50-64 MM FIXED) 60%/ RURAL (CBE 40-64) 40%* | 95% | 4,402 | 53,135,975 | 10,396,581 | 276,684 | 63,809,240 | 13,442 | 4,747 | Dominated |
| 85 | Stage I to IV treatment with triennial MIXED screening: URBAN (45-49 CBE) (50-69 MM FIXED) 60%/ RURAL (CBE 45-69) 40%* | 95% | 4,402 | 53,035,136 | 10,396,581 | 276,684 | 63,708,401 | 14,308 | 4,453 | Dominated |
| 86 | Stage I to IV treatment with triennial MIXED screening: URBAN (45-49 CBE) (50-64 MM FIXED) 60%/ RURAL (CBE 45-64) 40%* | 95% | 4,402 | 54,049,576 | 10,396,581 | 276,684 | 64,722,842 | 12,636 | 5,122 | Dominated |
| 87 | Stage I to IV treatment with triennial MIXED screening: URBAN (50-69 MM FIXED) 60%/ RURAL (CBE 50-69) 40%* | 95% | 4,402 | 54,650,856 | 10,396,581 | 276,684 | 65,324,121 | 11,884 | 5,497 | Dominated |
| 88 | Stage I to IV treatment with triennial MIXED screening: URBAN (50-64 MM FIXED) 60%/ RURAL (CBE 50-64) 40%* | 95% | 4,402 | 56,199,838 | 10,396,581 | 276,684 | 66,873,103 | 9,684 | 6,906 | Dominated |
| 89 | Stage I to IV treatment with most efficient triennial MIXED screening strategy (MIXED, 40-69)+ FNA* | 95% | 4,402 | 53,557,982 | 11,208,251 | 292,272 | 65,058,506 | 14,959 | 4,349 | Dominated |
| 90 | Stage I to IV treatment with most efficient triennial MIXED screening strategy (MIXED, 40-69)+ FNA +BPC | 95% | 4,402 | 53,539,583 | 12,511,232 | 518,783 | 66,569,598 | 14,961 | 4,450 | Dominated |
| 91 | Stage I to IV treatment with most efficient triennial FIXED/MOBILE screening strategy (FIXED/MOBILE, 45-69) + BPC | 95% | 4,402 | 54,804,394 | 14,726,156 | 577,237 | 70,107,788 | 16,658 | 4,209 | Dominated |
| 92 | Stage I to IV treatment with most efficient triennial FIXED/MOBILE screening strategy (FIXED/MOBILE, 45-69) + EPC | 95% | 4,402 | 56,548,695 | 15,013,508 | 577,237 | 72,139,440 | 16,660 | 4,330 | Dominated |
| 93 | Stage I to IV treatment with most efficient triennial FIXED/MOBILE screening strategy (FIXED/MOBILE, 45-69) + EPC + Trastuzumab stage I & II | 95% | 4,402 | 63,531,877 | 15,064,248 | 586,980 | 79,183,105 | 17,063 | 4,641 | Dominated |
| 94 | Stage I to IV treatment with most expensive screening strategy (annual, FIXED60%/MOBILE40%, 40-69 ) + EPC + trastuzumab (all stages) | 95% | 4,402 | 103,306,498 | 19,638,424 | 625,949 | 123,570,871 | 18,737 | 6,595 | 87,243 |

**ACER**: Average cost-effectiveness ratio compared to the do nothing-scenario (US$ per DALY averted). **ICER**: Incremental cost effectiveness ratio, ratio of additional cost per additional life-year saved when next intervention is added to a mix (additional US$ per additional DALY saved). **NA**: Not applicable because intervention is less cost-effective than others.

*These scenarios include Standard Palliative Care (SPC)

** In 20012 US$ (1 SOL = 0,384 US$)

*** DALYs, disability-adjusted life-years (age weighted, 3% discounted)
