# Supplementary material for: Cost-Effectiveness Analysis of Breast Cancer Control Interventions in Peru
Source: PLoS One. 2013 Dec 10;8(12):e82575. doi: 10.1371/journal.pone.0082575 (PMC3859673; doi:10.1371/journal.pone.0082575)
Supplement: Table S3 — Results of sensitivity analysis on average cost-effectiveness ratios (ACERs) of recommended interventions. (DOCX) [file pone.0082575.s004.docx]

**Table S3**. **Results of sensitivity analysis on average cost-effectiveness ratios (ACERs) of recommended interventions**

| **Sce-nario number (#)** | **Intervention scenarios** | **ACER** | **Case fatality rates (-25%-+25%)** | **Disability weights (-25%-+25%)** | **Alternative sources for current stage distribution*** | **Lower screening attendance (-25%)**** | **Lower screening sensitivity (-25%)**** | **Increased unit cost mammogram (+25%)** | **Increased unit cost FNA (+25%)** | **Increased transportation multiplyers (+25%)** |
| --- | --- | --- | --- | --- | --- | --- | --- | --- | --- | --- |
| 4 | Stage I treatment & relapse only | 5,406 | 4,759-10,019 | 4,659-6,438 | 3,330-3,957 | 5,406 | 5,406 | 5,406 | 5,406 | 5,880 |
| 85 | Stage I to IV treatment with triennial MIXED screening: URBAN (45-49 CBE) (50-69 MM FIXED) 60%/ RURAL (CBE 45-69) 40%* | 4,453 | 3,661-5,267 | 4,017-4,994 | 3,434-4,323 | 5,985 | 5,892 | 4,532 | 4,453 | 4,877 |
| 83 | Stage I to IV treatment with triennial MIXED screening: URBAN (40-49 CBE) (50-69 MM FIXED) 60%/ RURAL (CBE 40-69) 40%* | 4,295 | 3,560-5,050 | 3,885-4,802 | 3,334-4,173 | 5,676 | 5,597 | 4,371 | 4,295 | 4,693 |
| 89 | Stage I to IV treatment with most efficient triennial MIXED: URBAN (40-49 CBE) (50-69 MM FIXED) 60%/ RURAL (CBE 40-69) 40%*+ FNA* | 4,349 | 3,605-5,114 | 3,934-4,862 | 3,376-4,225 | 5,744 | 5,664 | 4,425 | 4,349 | 4,747 |
| 90 | Stage I to IV treatment with most efficient triennial MIXED: URBAN (40-49 CBE) (50-69 MM FIXED) 60%/ RURAL (CBE 40-69) 40%* + FNA + BPC | 4,450 | 3,688-5,232 | 4,025-4,974 | 3,454-4,323 | 5,869 | 5,787 | 4,525 | 4,450 | 4,848 |
| 67 | Stage I to IV treatment with triennial mammography screening (45-69 years) FIXED 60%/MOBILE 40%* | 4,125 | 3,484-4,787 | 3,754-4,578 | 3,250-4,014 | 5,158 | 5,143 | 4,307 | 4,125 | 4,506 |
| 91 | Stage I to IV treatment with most efficient triennial FIXED/MOBILE screening strategy (FIXED/MOBILE, 45-69) + BPC | 4,209 | 3,554-4,883 | 3,830-4,671 | 3,316-4,095 | 5,320 | 5,237 | 4,390 | 4,209 | 4,589 |
| 65 | Stage I to IV treatment with triennial mammography screening (40-69 years) FIXED 60%/MOBILE 40%* | 4,167 | 3,535-4,820 | 3,798-4,617 | 3,295-4,056 | 5,129 | 5,115 | 4,390 | 4,167 | 4,549 |
| 60 | Stage I to IV treatment with biennial mammography screening (40-64 years) FIXED 60%/MOBILE 40%*† | 4,507 | 3,831-5,206 | 4,110-4,990 | 3,569-4,387 | 6,228 | 6,203 | 4,795 | 4,507 | 4,906 |
| 59 | Stage I to IV treatment with biennial mammography screening (40-69 years) FIXED 60%/MOBILE 40%*† | 4,582 | 3,898-5,289 | 4,180-5,071 | 3,631-4,460 | 5,519 | 5,498 | 4,895 | 4,582 | 4,987 |
| 55 | Stage I to IV treatment with annual mammography screening (45-69 years) FIXED 60%/MOBILE 40%*† | 5,318 | 4,522-6,141 | 4,850-5,886 | 4,213-5,177 | 6,501 | 6,253 | 5,777 | 5,318 | 5,776 |
| 53 | Stage I to IV treatment with annual mammography screening (40-69 years) FIXED 60%/MOBILE 40%*† | 5,682 | 4,852-6,541 | 5,189-6,278 | 4,516-5,532 | 6,818 | 6,582 | 6,262 | 5,682 | 6,170 |
| 94 | Stage I to IV treatment with most expensive screening strategy (annual, FIXED60%/MOBILE40%, 40-69 ) + EPC + trastuzumab (all stages)† | 6,595 | 5,749-7,665 | 6,128-7,385 | 5,351-6,525 | 8,037 | 7,760 | 7,148 | 6,595 | 7,060 |

*Alternative current stage distribution 1, according to Schwartsmann et al. (4% stage I, 7% stage II, 18% stage III, 70% stage IV)[49]. Alternative current stage distribution 2, according to Groot et al. (9.4%stage I, 14.2%stage II, 58.0%stage III, 18.4%stage IV)[17].

** Changes in screening attendance and screening sensitivity result in altered stage distributions and these altered stage distributions were used in this analysis.
